# New Insights into c-Ret Signalling Pathway in the Enteric Nervous System and Its Relationship with ALS

**DOI:** 10.1155/2014/328348

**Published:** 2014-04-28

**Authors:** M. J. Luesma, I. Cantarero, J. M. Álvarez-Dotu, S. Santander, C. Junquera

**Affiliations:** ^1^Department of Human Anatomy and Histology, Faculty of Medicine, University of Zaragoza, Domingo Miral, s/n, 50009 Zaragoza, Spain; ^2^Aragon Health Sciences Institute (I+CS), Zaragoza, Spain; ^3^Department of Cellular Biology, Physiology and Immunology, Faculty of Medicine, University of Córdoba, Spain; ^4^Maimonides Institute for Biomedical Research of Cordoba (IMIBIC), Spain; ^5^Department of Obstetrics, Gynaecology and Surgery, University of Zaragoza, Spain; ^6^Department Pharmacology and Physiology, Faculty of Medicine, University of Zaragoza, Spain

## Abstract

The receptor tyrosine kinase Ret (c-Ret) transduces the glial cell line-derived neurotrophic factor (GDNF) signal, one of the neurotrophic factors related to the degeneration process or the regeneration activity of motor neurons in amyotrophic lateral sclerosis (ALS). The phosphorylation of several tyrosine residues of c-Ret seems to be altered in ALS. c-Ret is expressed in motor neurons and in the enteric nervous system (ENS) during the embryonic period. The characteristics of the ENS allow using it as model for central nervous system (CNS) study and being potentially useful for the research of human neurological diseases such as ALS. The aim of the present study was to investigate the cellular localization and quantitative evaluation of marker c-Ret in the adult human gut. To assess the nature of c-Ret positive cells, we performed colocalization with specific markers of cells that typically are located in the enteric ganglia. The colocalization of PGP9.5 and c-Ret was preferentially intense in enteric neurons with oval morphology and mostly peripherally localized in the ganglion, so we concluded that the c-Ret receptor is expressed by a specific subtype of enteric neurons in the mature human ENS of the gut. The functional significance of these c-Ret positive neurons is discussed.

## 1. Introduction

Amyotrophic lateral sclerosis (ALS) is a fatal neurodegenerative disease characterized by selective degeneration of motor neurons located in the spinal cord, brain stem, and motor cortex, resulting in progressive atrophy and paralysis of limb, bulbar, and respiratory muscles [1–5]. Glial cell line-derived neurotrophic factor (GDNF) has been thought to be one of the potent neurotrophic factors related to the degeneration process or the regeneration activity of motor neurons in ALS. In particular GDNF is a member of the transforming growth factor-*β* superfamily which promotes the survival of motor neurons and mesencephalic dopaminergic neurons [6, 7]. The GDNF signal is transduced by a specific receptor, the proto-oncogene product RET (a tyrosine kinase receptor c-Ret), in association with another receptor GDNFR-*α* (GFR*α*-1) [6, 8–12]. The phosphorylation of several tyrosine residues of RET is a crucial step in the intracellular signaling pathway [6, 13] and it seems to be altered in ALS [5]. c-Ret is expressed in the restricted tissues including motor neurons determining the specific distribution of GDNF-responsive neurons, [12]; one of these tissues is the enteric nervous system (ENS) where Ret is highly expressed during the embryonic period. It is a crucial signal for the development of enteric neurons [14–19].

On the other hand, the enteric nervous system (ENS) is a collection of neurons in the gastrointestinal tract that constitutes the “brain of the gut.” This system provides neural control for all functions of the gastrointestinal tract. Subsequent examination of the functional and chemical diversity of enteric neurons revealed that the enteric nervous system closely resembles the central nervous system (CNS). In fact, the ultrastructure of the ENS is more similar to the CNS ultrastructure than to the rest of the peripheral nervous system (PNS) [20, 21]. Interestingly, gut and brain can show a parallel pathology in most neurodegenerative diseases [22]. In connection with ALS, the tight association between ALS and ENS was firstly described by Sanchez and collaborators, when the generation of GDNF mutant mice led to massive loss of ENS neurons [23]. These characteristics lead researchers to develop ENS as model for CNS study and being potentially useful for the research of human neurological diseases such as ALS. Moreover, recent studies in an animal model of ALS (TDP-43 A315T transgenic mice) showed a lack of severe muscle atrophy or external muscle weakness in later stages of the disease although degeneration of myenteric neurons by the accumulation of TDP-43 was detected. Rather than severe muscle weakness, the progressively thinned colon contributed to the death of the transgenic mice [24]. For this reason, it would be interesting to study and provide additional information on the location of c-Ret in gastrointestinal human samples in order to extrapolate to ALS patients, for better understanding of the disease and the possible identification of target markers of disease.

The ENS arises from two regions of the neural crest: the vagal neural crest which gives rise to the vast majority of enteric neurons throughout the gastrointestinal tract and the sacral neural crest which contributes a smaller number of cells that are mainly distributed within the hindgut.

Neural crest cells (NCCs) migrate and differentiate into a variety of cell lineages such as melanocytes, neurons, glial cells, myofibroblasts, chondrocytes, and osteoblasts [25–28]. Some of the migrating NCCs are multipotent [29] and they maintain their nature at the time they reach the foregut mesenchyme [30–32]. Once within the gut, these enteric neural crest stem cells (eNCSCs) generate a vast array of phenotypically diverse neurons and glial cells. Furthermore, differentiation of eNCSCs is asynchronous and therefore multipotent progenitor cells coexist with differentiated cell types [30].

A number of markers have become available that label migratory or post- migratory NCCs within the gastrointestinal tract of embryonic mice and rats. Previous studies have shown that undifferentiated enteric crest-derived cells are Phox2b(+)/c-Ret(+)/p75(+)/Sox10(+) [33].

c-Ret is expressed during embryogenesis on various neuronal subsets of the central and peripheral nervous system and it is crucial for the development of sympathetic, parasympathetic, motor, and sensory neurons. Furthermore, c-Ret is necessary for the postnatal maintenance of dopaminergic neurons, but it also regulates also the development of the nervous plexuses of the entire intestinal tract [14, 18, 19, 34]. Mutations in c-Ret, albeit drastically, affect only a subset of peripheral nervous system ganglia. Thus, loss of c-Ret function in humans can lead to congenital megacolon (Hirschsprung's disease), a condition characterized by absence of enteric ganglia [35–37], which demonstrates its important role in the enteric neurogenesis.

Due to the fact that there are no immunohistochemical studies on the presence of c-Ret in the normal adult intestine, the aim of the present study was to investigate the cellular localization, distribution, and quantitative evaluation of c-Ret in the adult human intestine, using immunohistochemistry and immunofluorescence techniques in order to identify the cell types derived from neural crest that persist in the adult ENS. A double immunofluorescence procedure has been carried out to examine the colocalization of c-Ret marker with PGP9.5 (neuronal marker), with glial fibrillary acidic protein (GFAP) (glial marker), and with c-Kit (interstitial cell of Cajal marker).

## 2. Materials and Methods

The material included in our study was obtained from intestinal biopsies taken during endoscopic examination from 12 patients with different digestive pathologies (the average age was 46 years; range 31–68). None of the patients included in the study have bowel level affectation. The use of human tissues was approved by the Ethics Committee for Clinical Investigation of Aragon (CEICA; ICS08/0082), Zaragoza, Spain.

Intestinal biopsy specimens were fixed in 10% neutral buffered formalin, embedded in paraffin, cut, and processed for immunohistochemistry.

### 2.1. Immunohistochemistry

The intestinal samples were fixed for 6 hours with formol saline solution at 10% pH 7. Immunohistochemical staining was performed on paraffin sections with 5 µm of thickness using the immunohistochemistry EnVision (Dako) method. The primary antibody used in this study was the anti-Ret monoclonal antibody (clone RET01, dilution 1 : 50, Affinity BioReagents, ABR). This antibody was diluted with Dako diluent (S2022). The tissue sections were deparaffined in xylene (10 min twice) and rehydrated in a graded ethanol series up to distilled water. Prior to all assays, for heat-induced antigen retrieval, the samples were treated for 6 minutes in an 800 W microwave oven with a 10% citrate buffer (Dako S2031) in distilled water and at 360 W for 5 additional minutes. After washing twice with PBS, for 3 minutes, the sections were treated with endogenous peroxidase blocking (Dako S2001) for 20 minutes, washed in distilled water, and treated with the blocking buffer [100 mL PBS, 2 mL triton X100, 0.25 mL BSA (A4503 SIGMA)], for 3 minutes. The blocking was repeated for a second time. The sections were incubated with the primary antibody solution for 30 minutes followed by rinsing in PBS, for 3 minutes. The visualization was made by incubating with an Envision peroxidase-based visualization kit (Dako K5007) over a period of 30 minutes, washed twice in PBS, for 3 min according to manufacturer's directions. To confirm the presence of immunocomplexes, 3,3′-diaminobenzidine was used as chromogene and hydrogen peroxide as substrate. The samples were washed twice in distilled water, contrasted with Mayer's haematoxylin for 7 minutes, washed in tap water for 15 minutes, dehydrated in a graded series of ethanol, cleared in xylene, and coverslipped with DPX. Digital microscope images were captured by means of an Olympus BX 51 microscope and analyzed using public domain image software: ImageJ: Image Processing and Analysis in Java available from http://rsb.info.nih.gov/ij/.

### 2.2. Counting Methods

The immunolabelled neurons were quantified in a random intestinal sample of 3 cm in length from each patient. 30 areas per region were studied from each sample. The total number of ganglia (myenteric and submucosal ganglia) and the total number of the c-Ret positive cells, compared to total morphologically recognized neurons, were counted.

Cells were counted by two independent investigators. If the cell count differed by 10%, a third investigator then analyzed the samples. The final cell count was the mathematical average of the independent cell counts. To minimize errors, only those neurons in which the nucleus was clearly visible were confirmed as positive controls.

### 2.3. Immunofluorescence

The expression and distribution of proteins were studied by the indirect immunofluorescence staining method for anti-Ret monoclonal antibody (clone RET01, dilution 1 : 50, Affinity BioReagents, ABR), polyclonal rabbit anti-glial fibrillary acidic protein (GFAP, dilution 1 : 100, Dako), polyclonal rabbit anti-PGP 9.5 (dilution 1 : 350, Dako), polyclonal rabbit anti-human c-Kit (dilution 1 : 50, Dako), goat anti-mouse IgG (HL) Alexa Fluor 488 (dilution 1 : 1000; Molecular Probes Invitrogen), and goat anti-rabbit IgG (H+L) Alexa Fluor 546 (dilution 1 : 1000; Molecular Probes Invitrogen).

The antibodies were diluted in Dako (S2022) diluent. Tissue sections were deparaffined in xylene (10 minutes twice) and rehydrated in a graded ethanol series up to distilled water. Before all assays, for heat-induced antigen retrieval, the samples were treated during 6 minutes in an 800 W microwave oven with 10% citrate buffer (Dako S2031) in distilled water and at 360 W for 5 additional minutes. After washing three times with PBS, for 5 minutes, the sections were treated with blocking buffer [100 mL PBS, 2 mL triton X100, 0.25 mL BSA (A4503 SIGMA)] for 20 minutes. The blocking was repeated for a second time. Sections were incubated with the primary antibody solution for 20 hours followed by three rinses in PBS for 5 minutes. The visualization was made with Alexa Fluor for 90 minutes, and then the sections werewashed in PBS three times for 10 minutes and incubated with 5 µM of DRAQ5 (Biostatus, Leicestershire, UK) to counterstain the nuclei. Finally, cover slips were then mounted in Mowiol Sigma-Aldrich, Deisenhofen, Germany) and cell staining was documented using Leica TCS SL Laser Scanning Confocal Microscope.

### 2.4. Statistical Analysis

Data are expressed as mean ± SEM. Differences were analyzed by Student's *t*-test. *P* < 0.05 was considered significant (*), *P* < 0.01 very significant (**), and *P* < 0.001 extremely significant (***).

## 3. Results 

### 3.1. c-Ret Immunoreactivity in Myenteric and Submucosal Plexus

**Figure 1:**
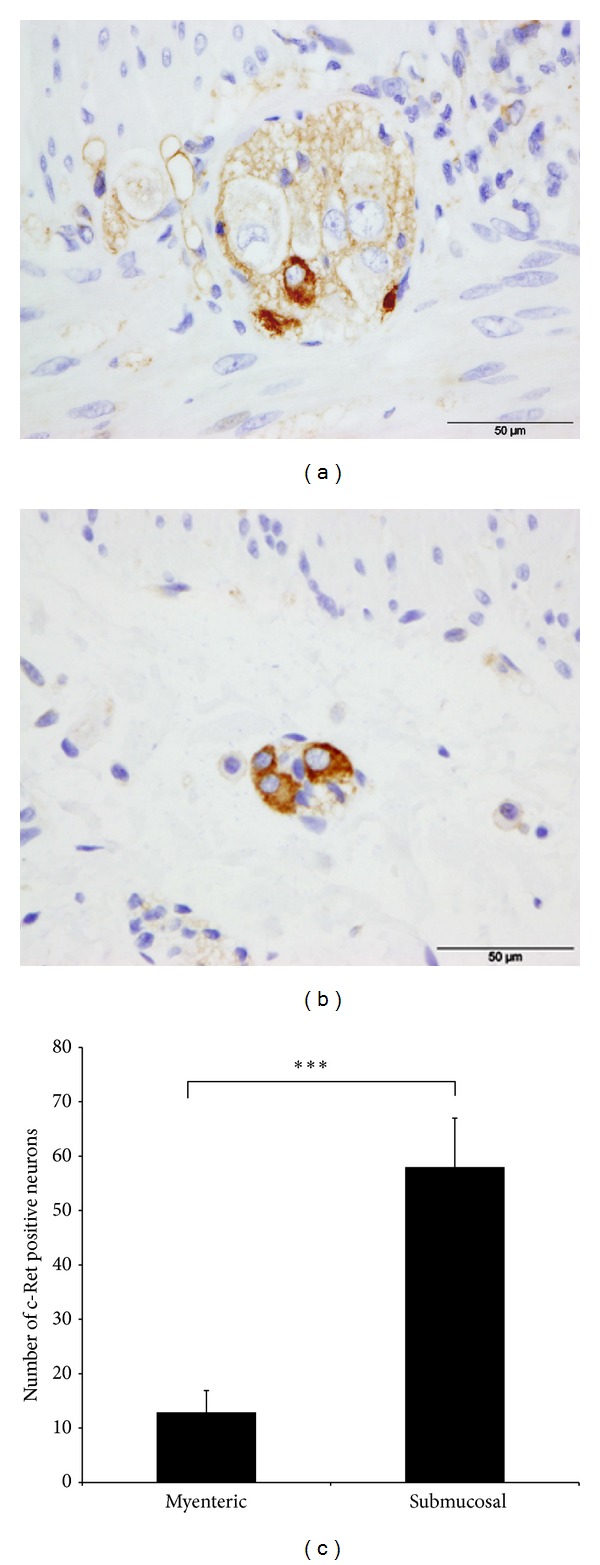
Immunocytochemical localization of the c-Ret receptor tyrosine kinase in the myenteric 1(a) and submucosal plexus 1(b). Human adult normal duodenum. Note that only the cytoplasm of some neurones appears labelled. 168 × 62 mm (300 × 300 DPI). Histogram showing percentage of Ret positive-neurons.  ***P = 0.001  by Student's *t* test 1(c).

Intense c-Ret immunoreactivity was detected in all cases studied of adult human intestine. c-Ret immunoreactivity was positive in the myenteric and submucosal ganglia, although significant differences between them were found. In the myenteric ganglia, only a few cells inside the ganglia were labelled (Figure 1(a)), while in the submucosal ganglia most cells were labeled (Figure 1(b)). On average, 32 myenteric ganglia (range 22–42) and 36 submucosal ganglia (range 19–54) per sample were counted. The total number of myenteric neurons counted in this study was 4.284, 12.9% of which showed c-Ret positivity. However, in the submucosal plexus, the total number of neurons counted was 1.486; 58% were c-Ret positive. There are significant differences between the two plexuses (Figure 1(c)).

### 3.2. PGP9.5, GFAP, and c-Kit Immunodetection in Adult Enteric Neurons

**Figure 2:**
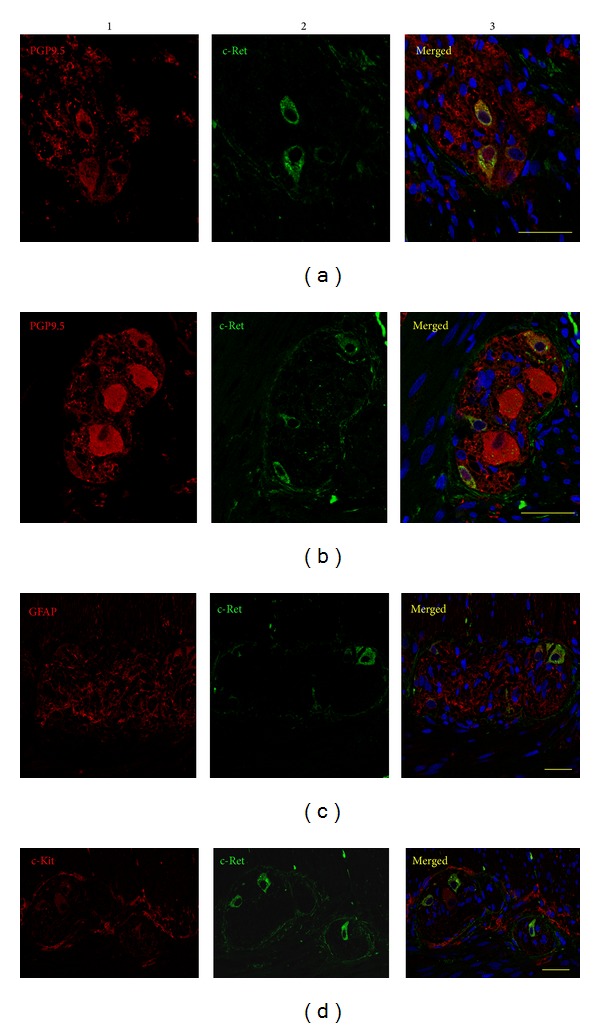
Confocal microscopy. PGP9.5-ir, GFAP-ir, and c-Kit-ir cells in column 1, rows (a) and (b), (c), and (d), respectively; c-Ret-ir in column 2 and merged images in column 3. Example of PGP9.5/c-Ret colocalization; adult enteric neurons labelled with PGP9.5 (a)-1, c-Ret (a)-2, and merged image (a)-3. However, this colocalization does not appear to always occur; PGP9.5-ir neurons (b)-1 does not colocalize with c-Ret neurons (b)-2 within the same ganglia (b)-3. GFAP-ir (c)-1 and c-Ret-ir cells (c)-2 are distinct cells (c)-3. Also, c-Kit-ir cells (d)-1 are c-Ret negatives (d)-2. Overlay image shows no colocalization between the two proteins (d)-3. Nuclear staining (blue). The scale bar is 20 µm in length and applies to all images. 168 × 228 (300 × 300 DPI).

We performed a double immunofluorescence technique to assess coexpression of c-Ret/PGP9.5, c-Ret/GFAP and c-Ret/c-Kit. PGP9.5-immunoreactive cells appeared within myenteric ganglia (Figures 2(a)-1 and 2(b)-1), GFAP-ir labelled cells appeared within myenteric ganglia and around neurons (Figure 2(c)-1), and c-Kit labelled cells appeared around myenteric ganglia (Figure 2(d)-1). In the adult intestine, some of c-Ret-positive cells also exhibited PGP9.5-ir. The colocalization of PGP9.5 and c-Ret was preferentially intense in enteric neurons with oval morphology and mostly peripherally localized in the ganglion (Figure 2(a)-3). In contrast, there was no colocation in other subpopulations of larger neurons with wide cytoplasm (Figure 2(b)-3). In neither case studied, did we observe colocalization with GFAP, glial cells marker (Figure 2(c)-3), and c-Kit, selective marker of Interstitial cells of Cajal (Figure 2(d)-3).

The pattern of c-Ret expression observed with optical and confocal microscopy was similar (Figures 2(a)-2, 2(b)-2, 2(c)-2, and 2(d)-2).

## 4. Discussion

The tyrosine kinase receptor c-Ret in association with another receptor GDNFR-*α* (GDNFR*α*-1) transduces GDNF mediated signal, which seems to be one of the neurotrophic factors related to ALS [6–12]. The phosphorylation of several tyrosine residues of c-Ret, a crucial step in the intracellular signal pathway [6, 13], is thought to be altered in ALS [5].

c-Ret is expressed during the embryonic period as a signal for the development of neurons in the enteric nervous system [12, 14–19]. There are no previous immunohistochemical studies to confirm the presence of c-Ret (neural crest marker) in the normal adult human gastrointestinal tract, which would be interesting as an extrapolated model to ALS patients.

An immunohistochemical study published was the only one to show the existence of c-Ret positive neurons in the myenteric plexus of adult rats. No positive neurons were described in the submucosal plexus [38].

Although some myenteric neurons in humans have been characterized based on morphology, projections, and neurochemical staining [39–41], the subtypes of neurons present in the ENS of any region of the adult human gastrointestinal tract have not yet been systematically catalogued [42]. These c-Ret positive neurons could be a specific neuronal subpopulation of the enteric ganglia, whose functional role should be studied. At this step we wonder about the potential function of these neurons in the ENS.

Given the additional fact that intestinal smooth muscle cells might even be a major source of GDNF, modulation and/or promotion of their own smooth muscle innervation in postnatal period might involve c-Ret receptors in adult enteric neurons [38]. However, a neuroprotective role (by mediating GDNF through the GFR*α*1/c-Ret receptor complex) as described for cerebellar Purkinje cells cannot be ruled out [43].

Sharkey and Parr (1996) revised the literature on this issue addressing the question about the enteric nature of these neurons, taking into account that these cells are postmitotic. These authors suggested that, since cell division in adult neurons was unlikely, the possible explanation for neuron addition in adulthood was a potential precursor nature of these cells in myenteric ganglia and their capability of late proliferation into adult neurons [44]. Although numerous experimental models have suggested the existence of neurogenesis in the adult ENS, these cells have not been identified in vivo [45–48]. These cells could in situ present immunohistochemical characteristics according to the embryonic origin of the neural crest. Therefore, our results suggested that the positive c-Ret neurons could answer this supposition.

On the other hand, Stemple and Anderson (1992) isolated postmigratory neural crest cells from fetal rat gut usingc-Ret and examined the developmental and proliferative capacities of these cells using a clonal culture system [49]. Many of the c-Ret positive cells divide symmetrically to generate small clones containing only neurons and consequently; they suggested that these c-Ret positive cells are committed neuronal progenitors [30].

Therefore, it has been demonstrated that during the tissue repair process, possible recapitulation of embrionary development takes place. Geuna et al. have suggested that NCCs, besides driving the development of many different tissue and organs during development, can play a role also in the postdevelopmental period of animal life because some of the migratory elements persist as a peripherical reserve pool of multipotent stem cells that can undergo late differentiation into mature cells [50].

We report immunohistochemical evidence for the presence of a group of adult enteric neurons stained with neural crest marker, c-Ret, in the normal adult duodenum. c-Ret was located in the myenteric and submucosal ganglia of the adult human duodenum and is expressed in some enteric neurons since it colocalized with pan-neuronal marker PGP9.5. Indeed, colocalization was more frequent in oval enteric neurons located peripherally in the ganglion, which suggested that c-Ret function is required not only during early foetal stages but also in the mature human ENS of the gut. The positivity to c-Ret may be indicative of different stages of neuronal maturation and, therefore, may be involved in adult enteric neurogenesis. We also suggest, like other authors, that multipotent NCCs coexist with differentiated cell types. Further studies are needed to study in depth the nature of these cells and their potential role in neurodegenerative diseases, such as ALS, could shed light on new molecular pathways possibly affected under neurodegenerative progression.

## References

[1] Rosen DR, Siddique T, Patterson D (1993). Mutations in Cu/Zn superoxide dismutase gene are associated with familial amyotrophic lateral sclerosis. *Nature*.

[2] Cleveland DW, Rothstein JD (2001). From Charcot to Lou Gehrig: deciphering selective motor neuron death in ALS. *Nature Reviews Neuroscience*.

[3] Rowland LP, Shneider NA (2001). Amyotrophic lateral sclerosis. *The New England Journal of Medicine*.

[4] Weiss MD, Weydt P, Carter GT (2004). Current pharmacological management of amyotropic lateral sclerosis and a role for rational polypharmacy. *Expert Opinion on Pharmacotherapy*.

[5] Ryu H, Jeon GS, Cashman NR, Kowall NW, Lee J (2011). Differential expression of c-Ret in motor neurons versus non-neuronal cells is linked to the pathogenesis of ALS. *Laboratory Investigation*.

[6] Mitsuma N, Yamamoto M, Li M (1999). Expression of GDNF receptor (RET and GDNFR-*α*) mRNAs in the spinal cord of patients with amyotrophic lateral sclerosis. *Brain Research*.

[7] Yamamoto M, Li M, Mitsuma N (2001). Preserved phosphorylation of RET receptor protein in spinal motor neurons of patients with amyotrophic lateral sclerosis: an immunohistochemical study by a phosphorylation-specific antibody at tyrosine 1062. *Brain Research*.

[8] GFRa Nomenclature Committee (1997). Nomenclature of GPI-linked receptors for the GDNF ligand family. *Neuron*.

[9] Takahashi M, Cooper GM (1987). Ret transforming gene encodes a fusion protein homologous to tyrosine kinases. *Molecular and Cellular Biology*.

[10] Takahashi M, Ritz J, Cooper GM (1985). Activation of a novel human transforming gene, ret, by DNA rearrangement. *Cell*.

[11] Treanor JJS, Goodman L, De Sauvage F (1996). Characterization of a multicomponent receptor for GDNF. *Nature*.

[12] Trupp M, Arenas E, Fainzilber M (1996). Functional receptor for GDNF encoded by the c-ret proto-oncogene. *Nature*.

[13] Ito Y, Yamamoto M, Li M (1998). Differential temporal expression of mRNAs for ciliary neurotrophic factor (CNTF), leukemia inhibitory factor (LIF), interleukin-6 (IL-6), and their receptors (CNTFR *α*, LIFR*β*, IL-6R *α* and gp130) in injured peripheral nerves. *Brain Research*.

[14] Pachnis V, Mankoo B, Costantini F (1993). Expression of the c-ret proto-oncogene during mouse embryogenesis. *Development*.

[15] Tsuzuki T, Takahashi M, Asai N, Iwashita T, Matsuyama M, Asai J (1995). Spatial and temporal expression of the ret proto-oncongene product in embryonic, infant and adult rat tissues. *Oncogene*.

[16] Durbec PL, Larsson-Blomberg LB, Schuchardt A, Costantini F, Pachnis V (1996). Common origin and developmental dependence on c-ret of subsets of enteric and sympathetic neuroblasts. *Development*.

[17] Trupp M, Arenas E, Fainzilber M (1996). Functional receptor for GDNF encoded by the c-ret proto-oncogene. *Nature*.

[18] Watanabe Y, Harada T, Ito T (1997). Ret proto-oncogene product is a useful marker of lineage determination in the development of the enteric nervous system in rats. *Journal of Pediatric Surgery*.

[19] Lucinia C, ’Angeloa LD, Patrunob M, Mascarellob F, de Girolamoa P, Castaldoa L (2011). GDNF family ligand RET receptor in the brain of adult zebrafish. *Neuroscience Letters*.

[20] Gershon MD, Kirchgessner AL, Wade PR, Johnson LR (1994). Functional anatomy of the enteric nervous system. *Physiology of the Gastrointestinal Tract*.

[21] Gershon MD (1997). Genes and lineages in the formation of the enteric nervous system. *Current Opinion in Neurobiology*.

[22] Natale G, Pasquali L, Paparelli A, Fornai F (2011). Parallel manifestations of neuropathologies in the enteric and central nervous systems. *Neurogastroenterology and Motility*.

[23] Sanchez MP, Silos-Santiago I, Frisen J, He B, Lira SA, Barbacid M (1996). Renal agenesis and the absence of enteric neurons in mice lacking GDNF. *Nature*.

[24] Guoa Y, Wanga Q, Zhanga K (2012). HO-1 induction in motor cortex and intestinal dysfunction in TDP-43 A315T transgenic mice. *Brain Research*.

[25] Anderson DJ (1997). Cellular and molecular biology of neural crest cell lineage determination. *Trends in Genetics*.

[26] Bronner-Fraser M (1995). Origins and developmental potential of the neural crest. *Experimental Cell Research*.

[27] Ito K, Sieber-Blum M (1993). Pluripotent and developmentally restricted neural-crest-derived cells in posterior visceral arches. *Developmental Biology*.

[28] Morrison SJ, White PM, Zock C, Anderson DJ (1999). Prospective identification, isolation by flow cytometry, and in vivo self-renewal of multipotent mammalian neural crest stem cells. *Cell*.

[29] Fraser SE, Bronner-Fraser M (1991). Migrating neural crest cells in the trunk of the avian embryo are multipotent. *Development*.

[30] Lo L, Anderson DJ (1995). Postmigratory neural crest cells expressing c-RET display restricted developmental and proliferative capacities. *Neuron*.

[31] Rothman TP, Le Douarin NM, Fontaine-Perus JC, Gershon MD (1990). Developmental potential of neural crest-derived cells migrating from segments of developing quail bowel back-grafted into younger chick host embryos. *Development*.

[32] Rothman TP, Goldowitz D, Gershon MD (1993). Inhibition of migration of neural crest-derived cells by the abnormal mesenchyme of the presumptive aganglionic bowel of ls/ls mice: analysis with aggregation and interspecies chimeras. *Developmental Biology*.

[33] Young HM, Bergner AJ, Müller T (2003). Acquisition of neuronal and glial markers by neural crest-derived cells in the mouse intestine. *Journal of Comparative Neurology*.

[34] Patel A, Harker N, Moreira-Santos L (2012). Differential RET signaling pathways drive development of the enteric lymphoid and nervous systems. *Science Signaling*.

[35] Parisi MA, Kapur RP (2000). Genetics of Hirschsprung disease. *Current Opinion in Pediatrics*.

[36] Manié S, Santoro M, Fusco A, Billaud M (2001). The RET receptor: function in development and dysfunction in congenital malformation. *Trends in Genetics*.

[37] Ishii K, Doi T, Inoue K (2013). Correlation between multiple RET mutations and severity of Hirschsprung’s disease. *Pediatric Surgery International*.

[38] Rodrigues DM, Li AY, Nair DG, Blennerhassett MG (2011). Glial cell line-derived neurotrophic factor is a key neurotrophin in the postnatal enteric nervous system. *Neurogastroenterology and Motility*.

[39] Porter AJ, Wattchow DA, Brookes SJH, Costa M (1997). The neurochemical coding and projections of circular muscle motor neurons in the human colon. *Gastroenterology*.

[40] Wattchow DA, Porter AJ, Brookes SJH, Costa M (1997). The polarity of neurochemically defined myenteric neurons in the human colon. *Gastroenterology*.

[41] Hens J, Vanderwinden J-M, De Laet M-H, Scheuermann DW, Timmermans J-P (2001). Morphological and neurochemical identification of enteric neurones with mucosal projections in the human small intestine. *Journal of Neurochemistry*.

[42] Hao MM, Young HM (2009). Development of enteric neuron diversity. *Journal of Cellular and Molecular Medicine*.

[43] Facello B, Castaldo L, De Martino L, Lucini C (2009). Glial cell line-derived neurotrophic factor in Purkinje cells of adult zebrafish: an autocrine mode of action?. *Neuroscience Letters*.

[44] Sharkey KA, Parr EJ (1996). The enteric nervous system in intestinal inflammation. *Canadian Journal of Gastroenterology*.

[45] Hanani M, Ledder O, Yutkin V (2003). Regeneration of myenteric plexus in the mouse colon after experimental denervation with benzalkonium chloride. *Journal of Comparative Neurology*.

[46] Ramalho FS, Santos GC, Ramalho LNZ, Kajiwara JK, Zucoloto S (1993). Myenteric neuron number after acute and chronic denervation of the proximal jejunum induced by benzalkonium chloride. *Neuroscience Letters*.

[47] Filogamo G, Cracco C (1995). Models of neuronal plasticity and repair in the enteric nervous system: a review. *Italian Journal of Anatomy and Embryology*.

[48] Liu M-T, Kuan Y-H, Wang J, Hen R, Gershon MD (2009). 5-HT4 receptor-mediated neuroprotection and neurogenesis in the enteric nervous system of adult mice. *Journal of Neuroscience*.

[49] Stemple DL, Anderson DJ (1992). Isolation of a stem cell for neurons and glia from the mammalian neural crest. *Cell*.

[50] Geuna S, Borrione P, Filogamo G (2002). Postnatal histogenesis in the peripheral nervous system. *International Journal of Developmental Neuroscience*.

